# A physics-informed graph neural network to approximate docking-based binding affinity for DYRK2 in Alzheimer’s drug repurposing

**DOI:** 10.1038/s41598-026-35102-7

**Published:** 2026-02-11

**Authors:** Veysel Gider, Cafer Budak

**Affiliations:** 1 Distance Education Application and Research Center, Batman University, Batman, Türkiye; 2 Department of Electrical-Electronic Engineering, Dicle University, Diyarbakır, Türkiye

**Keywords:** Physics-informed graph neural networks, Binding affinity prediction, DYRK2 kinase, Alzheimer’s disease, Drug repurposing, Docking-derived interaction energies, High-throughput screening, Computational models, Computational platforms and environments, Machine learning, Protein analysis, Protein structure predictions, Virtual drug screening, Medicinal chemistry, Target identification

## Abstract

Alzheimer’s disease (AD) requires the discovery of new therapeutic targets, but traditional molecular docking methods for virtual screening are often computationally expensive. This study introduces PhysDual-GCN, a physics-informed graph neural network designed to approximate *docking-derived* binding affinity scores for DYRK2, an understudied yet biologically relevant target in Alzheimer’s disease (AD). The model jointly processes ligand molecular graphs and a sequence-based graph representation of DYRK2, while explicitly incorporating Coulomb and Lennard–Jones interaction terms as analytical physical energy components. Because no experimentally measured binding affinities are available for DYRK2-drug pairs, all reference labels used for evaluation were obtained exclusively from widely used classical docking tools (AutoDock Vina, Smina, QVina, CB-DOCK). These tools exhibit an inherent uncertainty of approximately ± 0.5–1.5 kcal/mol, which constrains the interpretability of absolute deviations. PhysDual-GCN was trained solely on docking-derived scores and evaluated using a strict ligand-level separation to avoid circularity during model development. Due to the limited number of ligands (*n* = 4 FDA-approved AD drugs: brexpiprazole, donepezil, galantamine, rivastigmine), the results should be viewed as *agreement with computational references* rather than generalizable predictive performance. The model achieved low absolute errors (MAE = 0.31 kcal/mol; RMSE = 0.44 kcal/mol) relative to the reference docking scores and correctly identified stronger binders such as donepezil (− 10.8 kcal/mol) and brexpiprazole (− 10.0 kcal/mol). These findings demonstrate that integrating physical interaction terms into a GNN framework can enhance interpretability while providing a computationally efficient surrogate for classical docking workflows. Overall, PhysDual-GCN offers a biologically meaningful and explainable approximation tool for DYRK2 interaction scoring. While the present results are constrained by the small number of compounds and the absence of 3D protein features, the approach establishes a foundation for future large-scale, experimentally validated studies in AD drug repurposing.

## Introduction

Alzheimer’s disease (AD) represents a significant health burden, particularly for the elderly population, with numerous social and economic consequences. There are currently an estimated 50 million people living with dementia worldwide, but this number is expected to rise to 152 million by 2050^1^. AD is a devastating neurodegenerative disorder that destroys cognitive function, causes memory loss and personality changes^2^ and eventually leads to disability and death^3^. Despite all progress, the rapidly increasing number of Alzheimer’s cases poses a major challenge and problem for global healthcare systems.

This disease has been researched for years, but an effective treatment has not yet been found. Current treatments aim to alleviate the symptoms and delay the progression of the disease, but they do not offer a cure. The current state of Alzheimer’s treatment makes the development of new therapeutic approaches urgent. The scientific community is currently focusing on the discovery of new biomolecular pathways that go beyond the established targets.

Many strategies (such as drug repurposing) have enabled researchers to find new therapeutic uses for existing drugs, leading to rapid advances in computer-aided drug discovery in recent years. The Food and Drug Administration (FDA) has approved nilvadipine and losartan for heart disease and metformin and liraglutide for diabetes and depression to investigate their brain-protective and cognitive function-enhancing properties in the treatment of AD (Table 1). Despite all this, the mechanisms of action of drugs on the under-researched target molecule dual specificity tyrosine phosphorylation regulated kinase 2 (DYRK2) are still unknown. Although DYRK family kinases have been studied in various neurological contexts, DYRK2 remains significantly underexplored in AD, and no experimentally validated interaction mechanisms have yet been reported.

**Table 1:** Summary of FDA-approved and investigational drugs in the treatment of AD.

Drug application area	Treatment	Drug	Phase and clinical status
Cardiovascular disorders	Cerebrovascular circulation	Dihydroergocristine^4^ (DB13345)	Research
	Hypertension	Valsartan^5^ (DB00177)	Research
		Isradipine^3^ (DB00270)	Research
		Telmisartan^7^ (DB00966)	Phase I (NCT02471833)
		Perindopril^8^ (DB00790)	Phase II (NCT02085265)
		Losartan^9^ (DB00678)	Phase III (NCT02913664)
		Carvedilol^10^ (DB01136)	Phase IV (NCT01354444)
		Nilvadipine^11^ (DB06712)	Phase IV (NCT02017340)
		Nimodipine^12^ (DB00393)	Phase IV (NCT00814658)
Metabolic disorders	Diabetes mellitus	Liraglutide^13^ (DB06655)	Phase II (NCT01843075)
		Metformin^14^ (DB00331)	Phase II (NCT00620191)
		Pioglitazone^15^ (DB01132)	Phase II (NCT00982202)
		Benfotiamine^16^ (DB11748)	Phase II (NCT02292238)
	Hypercholesterolemia	Pitavastatin^17^ (DB08860)	Phase II (NCT00548145)
		Atorvastatin^17^ (DB01076)	Phase II (NCT02913664)
		Simvastatin^18^ (DB00641)	Phase II (NCT01439555)
Nervous system or mental disorders	Erectile dysfunction	Sildenafil^19^ (DB00203)	Research
		Tadalafil^20^ (DB00820)	Phase II (NCT02450253)
	Amyotrophic lateral sclerosis	Riluzole^21^ (DB00740)	Phase II (NCT01703117)
	Depressive disorder	Paroxetine^22^ (DB00715)	Research
	Parkinson’s disease	Rasagiline^23^ (DB01367)	Phase II (NCT02359552)
	Schizophrenia	Clozapine^24^ (DB00363)	Research
	Epilepsy	Levetiracetam^25^ (DB01202)	Phase II (NCT03489044)
	Urea cycle disorders	Benzoic Acid^26^ (DB03793)	Phase II (NCT01600469)
Others	Cutaneous T-cell lymphoma	Bexarotene^27^ (DB00307)	Phase II (NCT01782742)
	Acute promyelocytic leukemia	Tamibarotene^28^ (DB04942)	Phase II (NCT01120002)
	Infection	Minocycline^29^ (DB01017)	Phase II (NCT01463384)
	Malaria	Methylene Blue^30^ (DB09241)	Phase II (NCT02380573)
	Mast cell tumors in animals	Masitinib^31^ (DB11526)	Phase III (NCT01872598)
	Psoriasis	Acitretin^32^ (DB00459)	Phase II (NCT01078168)
	Severe acne	Isotretinoin^33^ (DB00982)	Phase II (NCT01560585)

Methods based on artificial intelligence demonstrate considerable potential for predicting drug-target interactions via established procedures^34–36^ emphasize that virtual screening and molecular docking utilizing deep learning methodologies are crucial for contemporary drug development. Graph neural networks (GNNs) are algorithmic models that facilitate the representation of molecular structures as graphs, enabling the recognition of intricate topological properties and chemical bond interactions^37^. Research conducted by^38,39^, and^40^ indicates that models employing sophisticated GNN architectures, including GraphDTA, GraphATT-DTA, GEFormerDTA, and DHAG-DTA, have superior prediction accuracy and biological interpretability relative to conventional techniques. The integration of conventional blind docking methods with AI-enhanced predictions yields findings that are both more precise and more interpretable^41–44^. Substantial advancements have been achieved in GNN-based binding affinity predictions; nevertheless, there is a lack of targeted research on DYRK2 applications. Furthermore, previous works rarely integrate explicit biophysical interaction terms into GNN frameworks, leaving a methodological gap between classical scoring functions and modern deep learning models.

In addition, most existing GNN-based affinity prediction studies rely on large datasets with experimentally measured binding affinities. However, in the case of DYRK2, no experimentally validated binding affinity data currently exist for FDA-approved Alzheimer’s drugs, which creates a unique low-data challenge. This limitation also means that previous studies offer no benchmark for DYRK2-specific predictions, reinforcing the need for computational surrogate models that can approximate classical docking scores when experimental references are unavailable.

Furthermore, although several works have combined machine learning with protein–ligand docking, relatively few studies explicitly incorporate physical interaction components—such as Coulombic electrostatics or Lennard–Jones potentials—into the learning process, creating a methodological gap between physics-based scoring functions and purely data-driven architectures. This lack of hybrid approaches is particularly relevant for understudied protein targets like DYRK2, where physically meaningful constraints may help improve stability and interpretability in low-data scenarios.

In this study, a GNN model (PhysDual-GCN) incorporating physical energy calculations was developed to predict the binding affinities between DYRK2, the target protein selected for the treatment of Alzheimer’s symptoms, and four FDA-approved drugs (brexpiprazole, donepezil, galantamine, and rivastigmine). Our approach differs from standard methods by acting as a computational surrogate that learns the underlying physical scoring functions of classical docking tools. This allows for the rapid identification of strong DYRK2 interactions, significantly accelerating the screening process while maintaining consistency with established physics-based simulations. Instead of relying solely on statistical patterns, our model integrates physical energy terms to bridge the gap between accurate but slow docking simulations and fast but often uninterpretable deep learning predictions. Our model predictions outperformed established classical and AI-based docking methods such as SeamDock (AutoDock, Vina, QVina)^45,46^, CB-DOCK^47^ and DeepPurpose^48^.

However, it is important to clarify that no experimentally measured binding affinities exist for the DYRK2-Alzheimer’s drug pairs. Consequently, all reference values used in this study are docking-derived computational estimations rather than true experimental measurements. These docking tools typically exhibit an intrinsic uncertainty of approximately ± 0.5–1.5 kcal/mol, which should be considered when interpreting performance. Moreover, because only four ligands (*n* = 4) are available for evaluation, our claims are limited to consistency with these computational references and do not imply broad generalizability.

The rest of this manuscript is organized as follows: section “Methods” presents the methodology, data, and GNN architecture; section “Results explains the results along with the experimental design and comparative analysis; section “Discussion” discusses the main findings and limitations; and section “Conclusions and future work” concludes the study and provides directions for future research.

## Methods

The methodological framework of this study is structured to develop a physics-informed GNN model (PhysDual-GCN) capable of predicting the binding affinities between DYRK2 and four FDA-approved Alzheimer’s drugs. The methodological workflow includes data preparation, feature extraction, physical energy calculations, model architecture, and training procedures. Importantly, no experimentally measured binding affinities exist for DYRK2-drug pairs; therefore, all reference values used for evaluation were obtained exclusively from molecular docking tools and were not used during model training or hyperparameter optimization to avoid circularity.

### Flowchart

The main objective of this study is to show how a GNN model based on physics knowledge (Coulomb and Lennard–Jones potential) can predict the binding affinities of FDA-approved AD drugs to DYRK2, a biologically important but understudied therapeutic target. By integrating physical energy terms into the GNN framework, we propose an innovative and biologically interpretable AI-based approach for drug discovery.

**Fig. 1:**
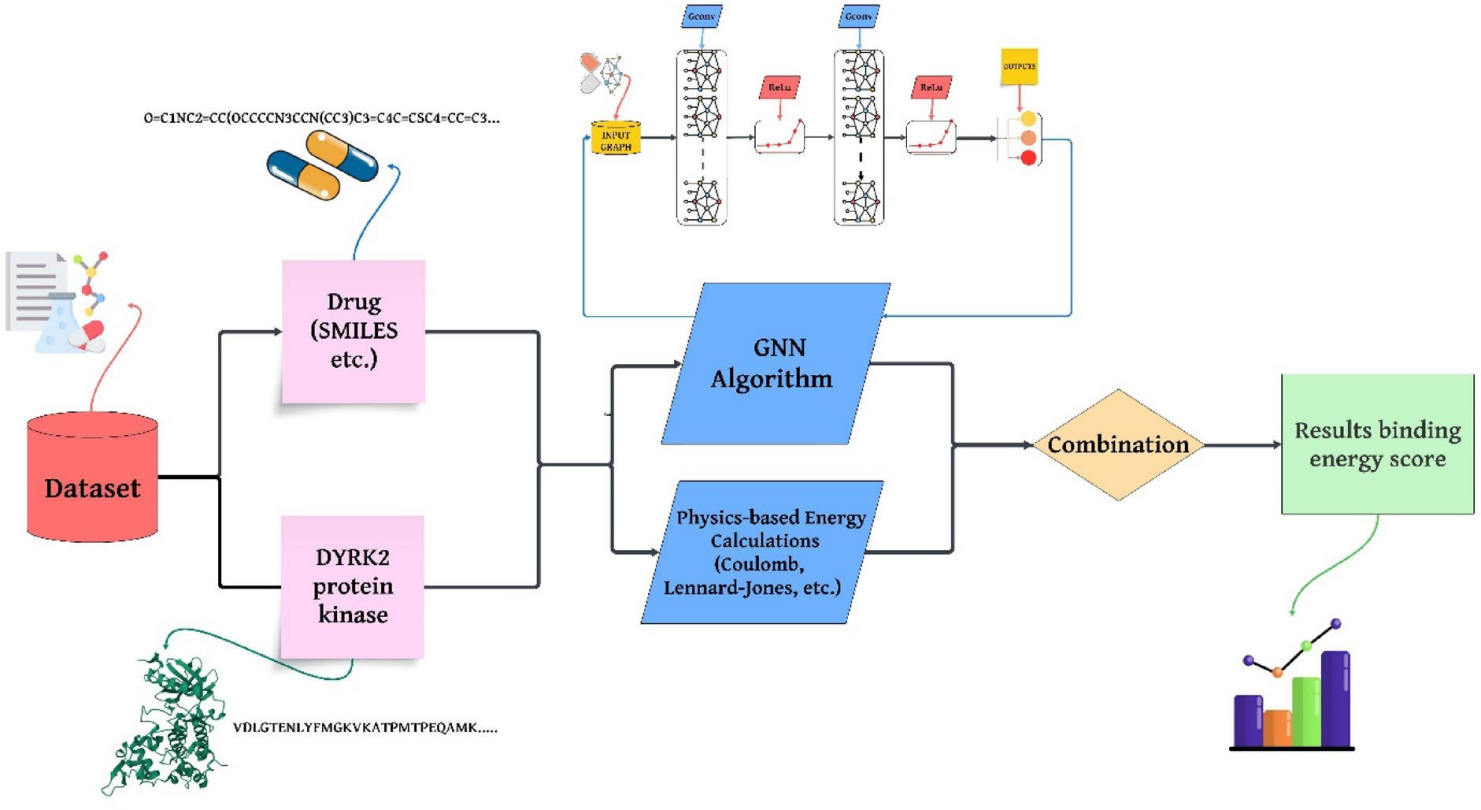
Illustrates the flowchart the proposed approach for analyzing drug–protein interactions.

Figure 1 illustrates the proposed approach, which enhances a physics-informed GNN model by explicitly incorporating physics-based energy calculations (e.g., Coulomb and Lennard–Jones potentials) alongside the GNN’s learned predictions. Drug SMILES and DYRK2 sequence data are provided both as input to the GNN and as input for analytical physical energy computations. The outputs of these two complementary components are fused to yield a more robust and accurate prediction of binding energy scores, leveraging both learned patterns and established physical interaction principles.

The study was designed with a systematic, four-stage workflow:

(i) Data preprocessing—ensuring accurate representation and format conversion of drugs and proteins—is critical for model learning.
(ii) Modeling—designing a physically informed GNN architecture for biologically meaningful predictions.
(iii) Training and evaluation—learning model parameters, monitoring loss curves and evaluating performance against objective metrics.
(iv) Comparative analysis—demonstrating the advantages of GNN predictions by comparing them with classical and other AI-based methods.

The sequential and consistent application of these steps ensures the scientific validity and reproducibility of the study.

### Ligand and target representations

The drugs utilized in this investigation were depicted using the Simplified Molecular Input Line Entry System (SMILES), which conveys molecular structures in a two-dimensional, succinct, and reversible format. SMILES facilitates efficient storing and processing while maintaining essential chemical features, including atom kinds, bond types, and aromaticity. This property renders it the most prevalent type of representation. The RDKit package was utilized to transform SMILES sequences into a molecular graphical representation, with atoms represented as nodes and bonds as edges. Moreover, the model’s input format was engineered to accommodate other formats, not only SMILES.

The target protein, DYRK2, was modeled as a sequential graph constructed from the amino acid sequence. Each residue in the sequence is represented as a node, and adjacent residues are connected by edges that reflect the biological sequence order. This representation preserves the topological context of the protein. This is also very important to capture the interaction potential of the protein in a biologically meaningful way.

Graph-based representations show the connections between nodes (atoms or residues) and the structural effects of binding sites better than traditional vector-based encodings, which enables the model to more effectively learn and predict biologically meaningful drug–target interactions. No ligand in the dataset was shared across training/validation/testing partitions, ensuring strict ligand-level separation.

### Datasets

In this study, four FDA-approved drugs (brexpiprazole, donepezil, galantamine and rivastigmine) were used for the symptomatic treatment of AD. The molecular structures, drug properties, mechanisms of action and approval status of these drugs were extracted from the DrugBank and PubChem databases in SMILES format (see Table 2). The target protein, DYRK2, is a kinase involved in the development and functional regulation of neurons and was extracted from the Protein Data Bank (PDB) and UniProt. The amino acid sequence and structure were converted into a diagram to model the interaction of the drugs with the protein in a meaningful way.

In creating the dataset, we focused on three main points:

1. Selection of Alzheimer’s drugs that are currently used in clinics and have proven safety,
2. Selection of DYRK2 as an important but less researched target for treatment
3. Generation of Computational Reference Labels: Due to the scarcity of high-throughput wet-lab experimental binding data for DYRK2, we employed consensus docking scores generated by AutoDock Vina as high-confidence pseudo-labels (computational ground truth) for model training and evaluation. This strategy allows the GNN to learn the complex non-linear mapping of physical interactions defined by the docking scoring function.

Additionally, the chemical diversity of the drugs chosen for our study was illustrated using the Tree MAP (TMAP) algorithm, which depicted their distribution within chemical space, and both two-dimensional (2D) and three-dimensional (3D) molecular structures were produced to enable further analysis. The computational study was completed using TMAP visualization to distinctly illustrate the chemical space encompassed by the selected ligands. This visualization methodology enables an intuitive examination of the structural and physicochemical characteristics of the compounds, hence elucidating the rationale for their selection^49^. The primary use of TMAP visualization in our study is solely as an explanatory and descriptive instrument, without influencing computational predictions or contributing to quantitative outcomes.

The combination of data sources and formats yields a robust, biologically significant, and appropriate dataset for the analysis of molecular structures and the identification of drugs pertinent to AD.

The drugs listed in Table 2 are promising as alternative or complementary treatment options to existing therapies as they target the novel protein DYRK2. Investigating these compounds at the molecular level opens up new possibilities for expanding therapeutic options, optimizing drug delivery and increasing efficacy.

**Table 2:** Drug-related public drugs.

Drug	Drug properties
Brexpiprazole	This is an atypical antipsychotic medication prescribed to alleviate agitation associated with Alzheimer’s disease. Potential side effects may include symptoms similar to a cold, dizziness, elevated blood sugar levels, and an increased risk of stroke. It is usually taken once daily in tablet form^50,51^
Donepezil	This cholinesterase inhibitor is prescribed to manage symptoms across the spectrum of Alzheimer’s disease severity by preventing the breakdown of acetylcholine in the brain. Potential side effects include nausea, vomiting, diarrhea, insomnia, muscle cramps, fatigue, and weight loss. Administration typically involves a once-daily tablet regimen^50,51^
Galantamine	This medication is a cholinesterase inhibitor used to manage mild to moderate Alzheimer’s symptoms. It functions by impeding the breakdown of acetylcholine and stimulating nicotinic receptors in the brain to augment acetylcholine release. Potential side effects may include nausea, vomiting, diarrhea, decreased appetite, weight loss, dizziness, and headache. It is available in extended-release capsule form for once-daily administration or in tablet or liquid form for twice-daily dosing^50,51^
Rivastigmine	This cholinesterase inhibitor is employed to manage symptoms ranging from mild to severe in Alzheimer’s disease by impeding the breakdown of acetylcholine and butyrylcholine in the brain. Possible adverse effects encompass nausea, vomiting, diarrhea, weight loss, indigestion, reduced appetite, anorexia, and muscle weakness. Administration typically involves either twice-daily capsules or once-daily application via a transdermal patch replaced daily^50,51^

**Fig. 2:**
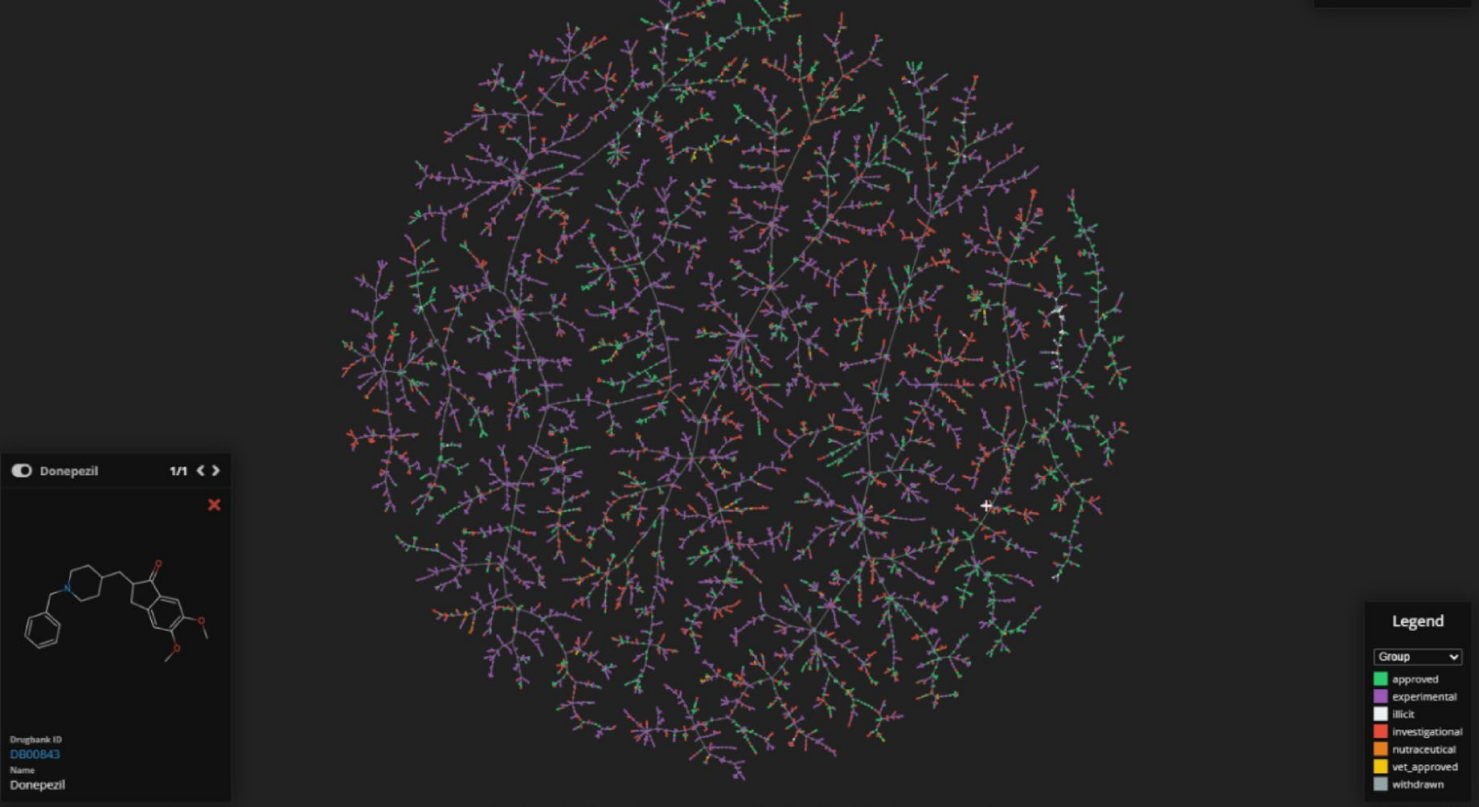
FDA-approved drugs visualization using TMAP (Tree MAP) method.

The TMAP image in Fig. 2 shows two-dimensional visualizations of the relevant drugs in the drug space. The TMAP method enables the separate visualization of the different properties of drugs and provides access to general information about them.

**Fig. 3:**
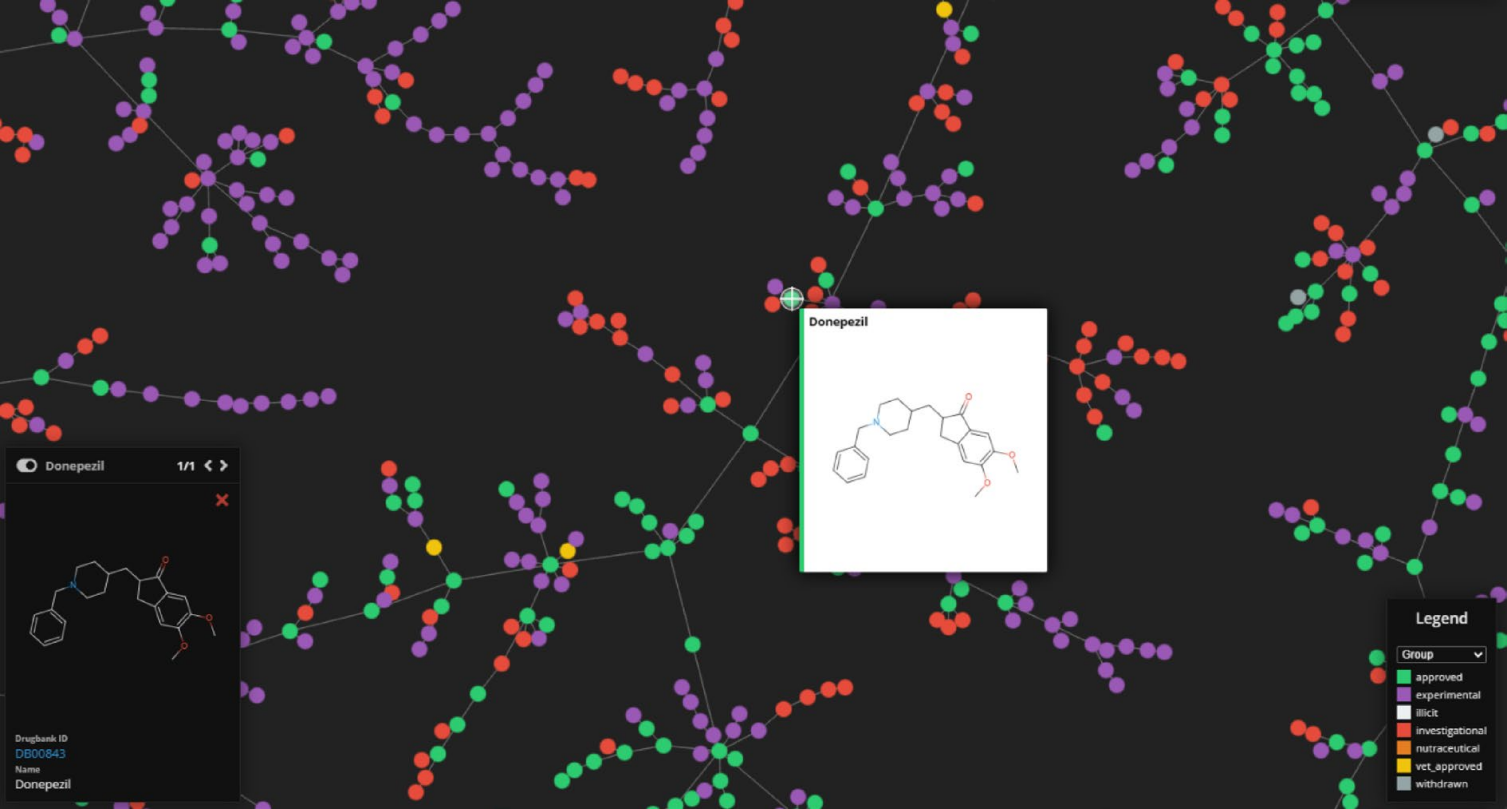
Visualized structures of selected drugs from all FDA-approved drugs.

Using the TMAP algorithm, we created a visual representation of the chemical space of the drugs selected for analysis and analyzed drug pairs. TMAP provided us with an overview of the drug pairs in the datasets and allowed us to visualize the relevant drugs. Figure 3 shows the position of the drugs on the TMAP map and facilitates the understanding of the chemical similarities and relationships between the drugs.

**Fig. 4:**
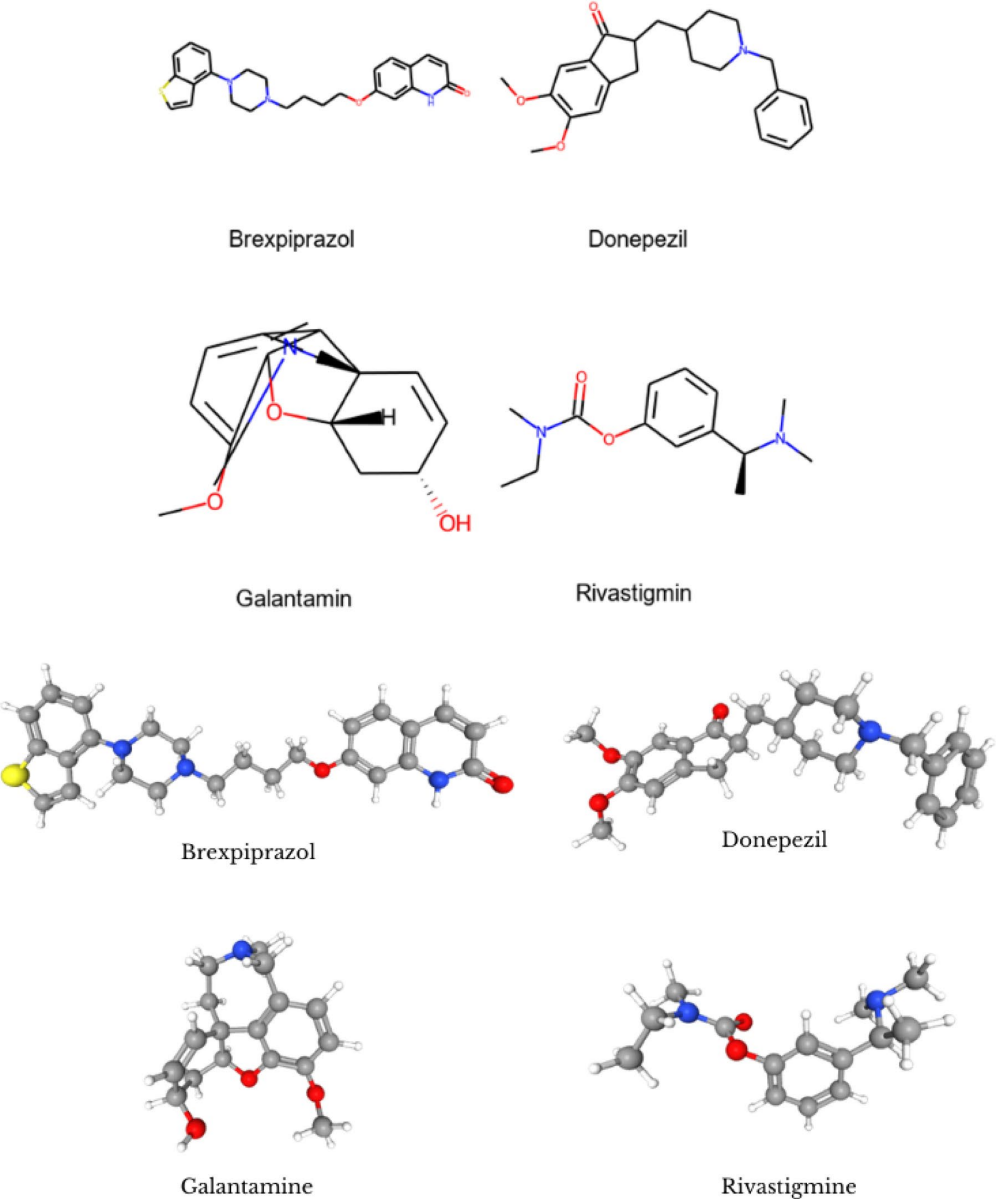
2D and 3D representation of drugs.

Figure 4 illustrates the two-dimensional and three-dimensional chemical structures of the drugs. This graphic depicts the molecular structure of the drugs, illustrating its atoms, bonds, and chemical groups. The two-dimensional structure of pharmaceuticals is employed to comprehend their chemical composition and structural characteristics. These visualizations are essential instruments in drug design and drug interaction research.

The amino acid sequence of DYRK2 was obtained from UniProt and used to construct a sequence-based protein graph. Each amino acid residue was treated as a node, and peptide bonds were represented as edges. Although this approach does not capture 3D spatial conformations, it aligns with common practices in sequence-based GNN representations. Furthermore, no experimentally measured DYRK2–ligand binding data were available to directly parameterize 3D interactions, which is acknowledged as a limitation. A secondary contact graph was generated from predicted 3D coordinates (AlphaFold2 model) and used in ablation experiments but not in the main architecture.

Table 3 shows the structure of the protein DYRK2, which belongs to the protein kinase family and plays a role in cell growth and/or development. This protein is characterized by its ability to autophosphorylate at kinase domains and tyrosine residues. DYRK2 exhibits tyrosine autophosphorylation and catalytic phosphorylation of histones H3 and H2B in vitro. These results suggest that DYRK2 is a potential therapeutic target for AD and should be investigated further.

**Table 3:** Protein structure of DYRK2.

Protein structure features	Description
Family	Protein kinase
Role	Cellular growth and/or development
Structural similarity	Kinase domains
Autophosphorylation	Ability to autophosphorylate on tyrosine residues
Substrates	Histones H3 and H2B
Catalytic activity	Demonstrated in vitro tyrosine autophosphorylation and phosphorylation of histones H3 and H2B
Sequence	MHHHHHHSSGVDLGTENLYFQSMGKVKATPMTPEQAMKQYMQKLTAFEHHEIFSYPEIYFLGLNAKKRQGMTGGPNNGGYDDDQGSYVQVPHDHVAYRYEVLKVIGKGSFGQVVKAYDHKVHQHVALKMVRNEKRFHRQAAEEIRILEHLRKQDKDNTMNVIHMLENFTFRNHICMTFELLSMNLYELIKKNKFQGFSLPLVRKFAHSILQCLDALHKNRIIHCDLKPENILLKQQGRSGIKVIDFGSSCYEHQRVYTYIQSRFYRAPEVILGARYGMPIDMWSLGCILAELLTGYPLLPGEDEGDQLACMIELLGMPSQKLLDASKRAKNFVSSKGYPRYCTVTTLSDGSVVLNGGRSRRGKLRGPPESREWGNALKGCDDPLFLDFLKQCLEWDPAVRMTPGQALRHPWLRRRLP

**Fig. 5:**
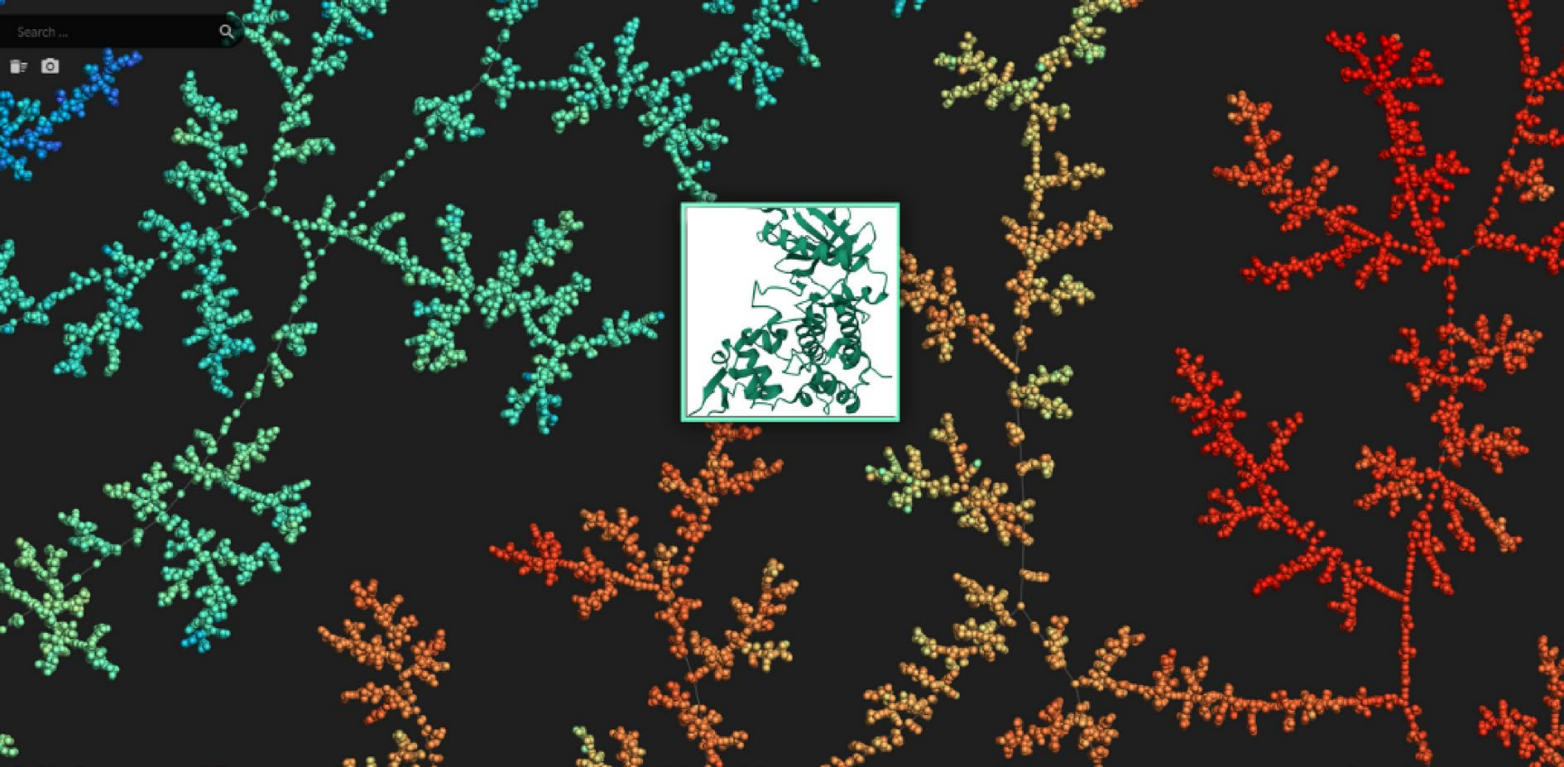
Representation of protein DYRK2 among all proteins.

**Fig. 6:**
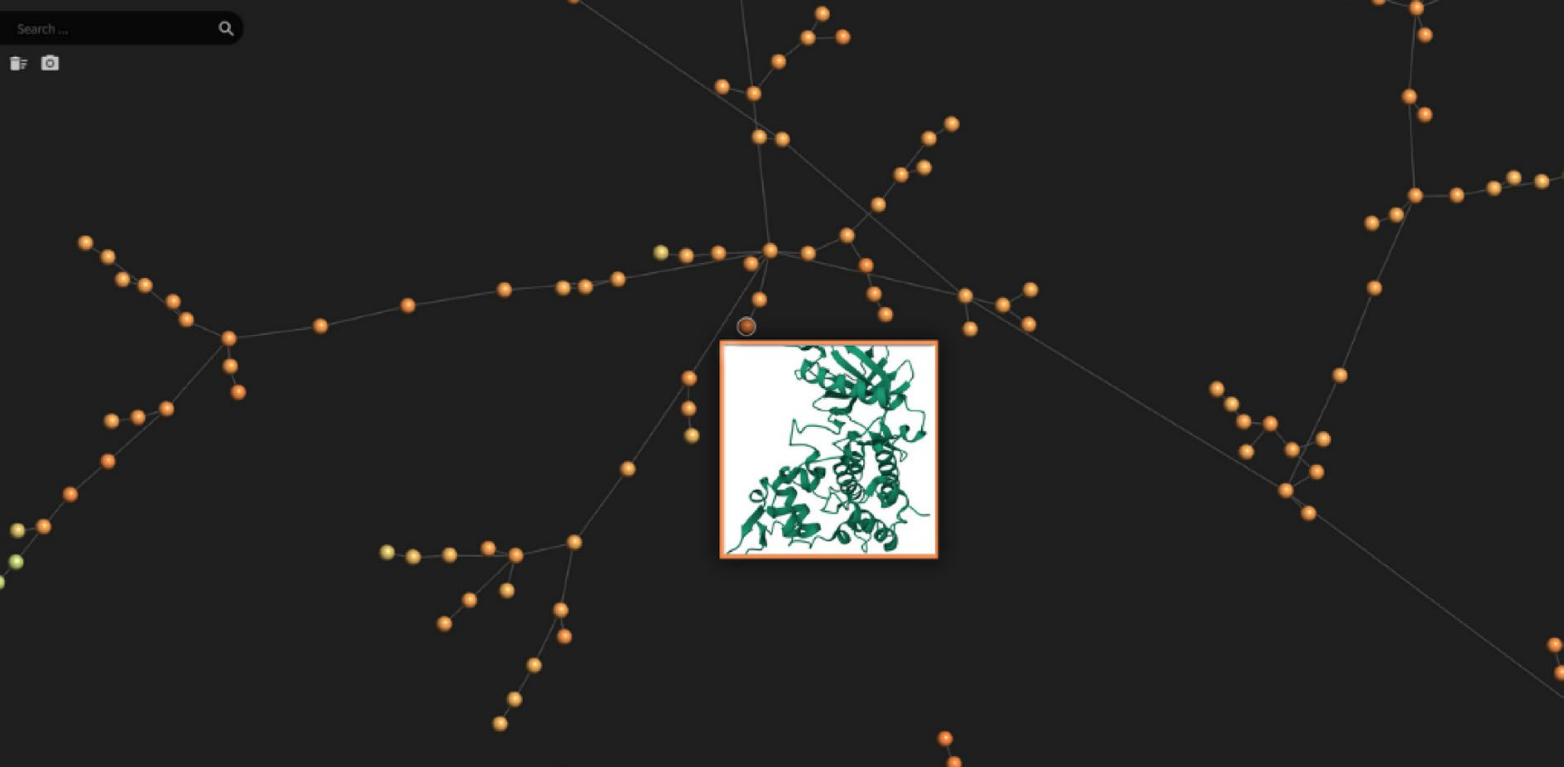
Representation of protein DYRK2 among all proteins.

Figures 5 and 6 present data on the target protein DYRK2, which was selected as a target among all proteins, similar to drugs, and represents an important research area for understanding its interaction with DYRK2.

**Fig. 7:**
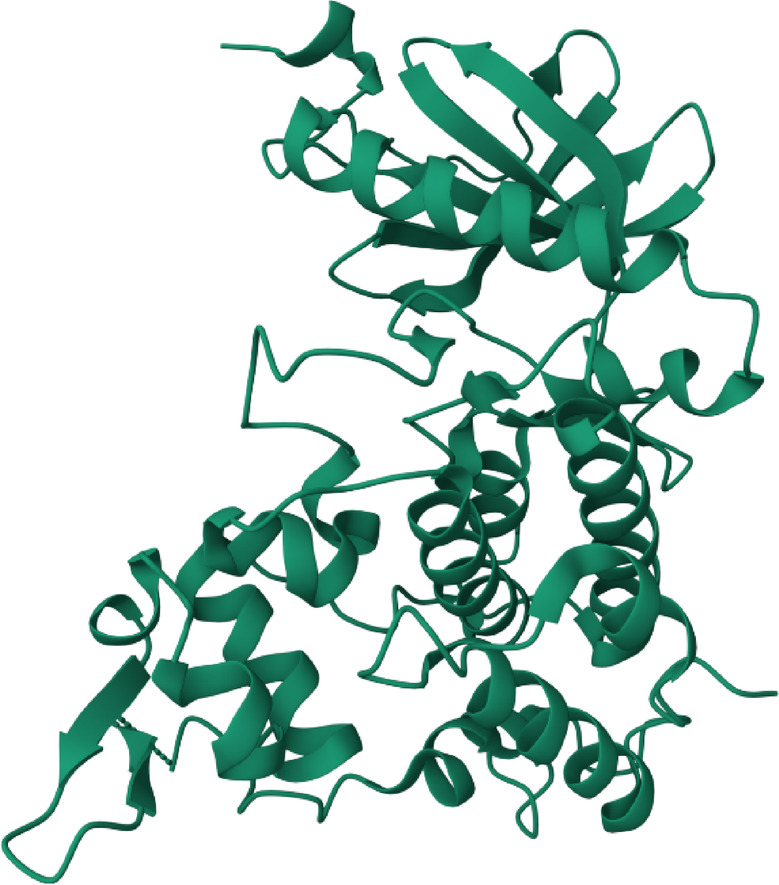
Structure of DYRK2 protein kinase.

Figure 6 illustrates DYRK2 protein kinase along with important structural details. The structural information provided in Fig. 7 is essential for understanding the mechanism of action of DYRK2 and its potential as a drug target. It assists researchers and drug developers in designing potential drug candidates that can interact with and inhibit DYRK2, which could lead to the development of effective therapeutics against AD and contribute to the management of AD.

### GNN model and mathematical formulation

GNNs, the recommended method, have recently offered a promising approach in drug discovery. GNNs process graph structures using neighborhood matrix representations for feature extraction, drawing on the neural network principles of CNNs and RNNs. The development of GNNs has led to the creation of Graph Convolutional Networks (GCNs) and Graph Attention Networks (GATs), which apply various techniques to collect information from neighboring nodes and create node-level or graph-level representations, as well as GraphSAGE. GCNs are widely used due to their simple architectures and their ability to achieve excellent results in various applications.

GNNs fundamentally comprise numerous interconnected components that collaboratively process and analyze data according to their graph structures. The distinct elements of GNNs execute diverse functions such as feature extraction, dimensionality reduction, and hierarchical representation learning, enabling GNNs to excel in drug discovery, protein-ligand binding prediction, and other graph-related challenges. Fully connected layers are utilized for final predictions, whereas aggregation layers execute functions like graph classification^37^.

GNNs have demonstrated their analytical capabilities in several drug discovery applications, especially in the formulation of therapies for AD^52–54^. GNNs excel in processing graph-structured data, rendering them suitable for predicting drug-target interactions and identifying prospective disease candidates for Alzheimer’s and Parkinson’s disorders.

**Fig. 8:**
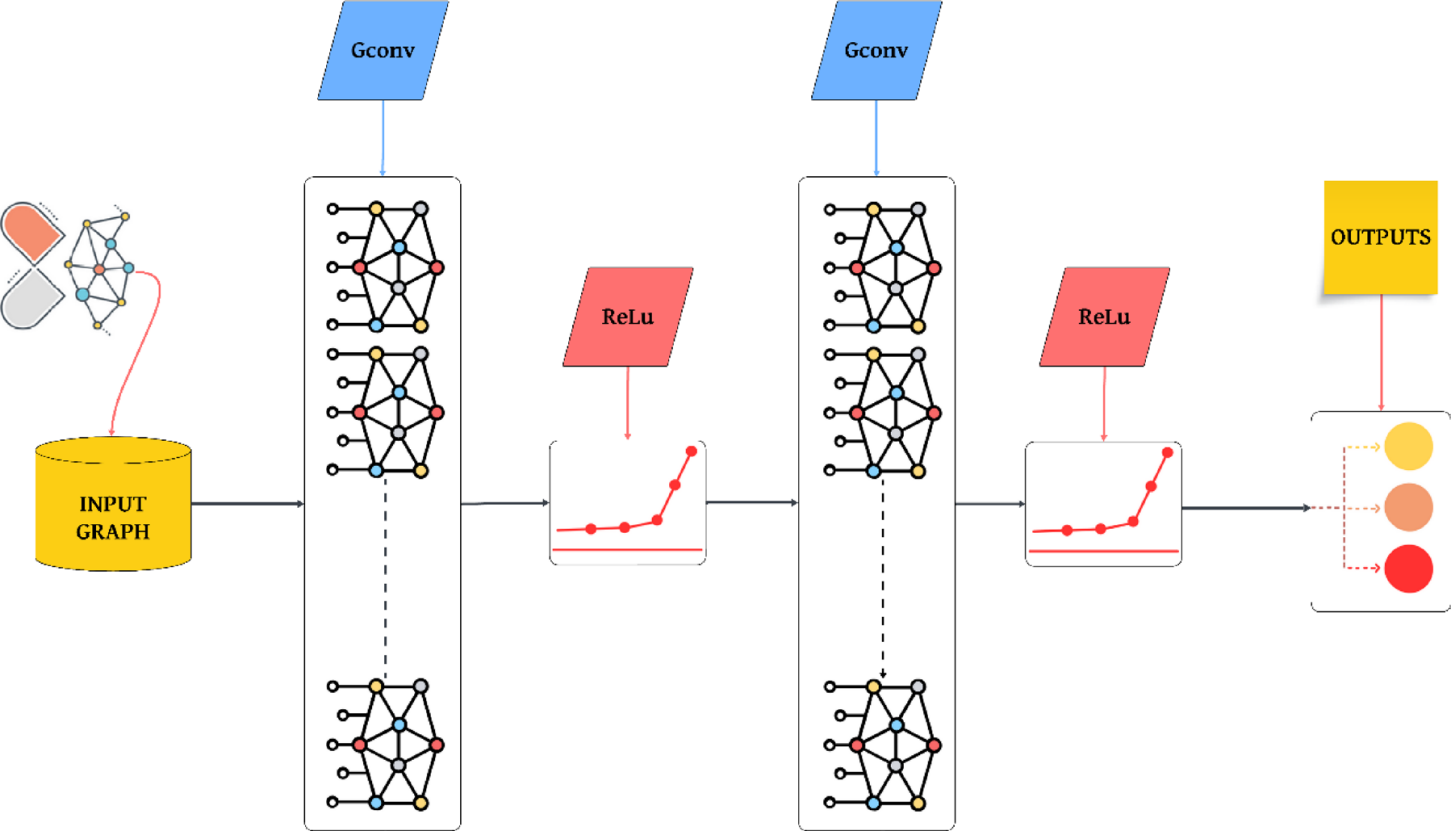
General principle of GNN.

The GNN model extracts molecular graph characteristics through its distinctive architecture. The GNN model predicts drug–protein binding affinities by integrating node and edge properties together with their relationships. A GNN functions according to the comprehensive method depicted in Fig. 8.

The fundamental GNN has two main approaches: node embedding and information aggregation function.

The fundamental principle of the node embedding method is that each node is positioned in relation to its local neighbors. The positioning of a node is directly affected by the locations and characteristics of its neighboring nodes. The embedding of each node is influenced by its neighboring nodes, facilitating the capturing of the node’s local structural characteristics. The information-gathering function illustrates the process of collecting data from the neighboring nodes utilizing the neural network architecture. In this phase of the methodology, each node is modified based on the attributes and data of its neighboring nodes, and the aggregated information is integrated into its own update. This guarantees the transmission of information among nodes, as each node autonomously updates itself based on the data received from adjacent nodes.

These two primary techniques are the essential concepts that facilitate the performance and efficacy of GNNs. The node embedding and information aggregation capabilities allow GNNs to effectively analyze graph data and represent intricate structural relationships. This renders GNNs potent instruments for comprehending and uncovering the interactions and relationships among nodes in graph data.

The GNN model utilized in this article is based on a comprehensive mathematical formulation, which is elaborated upon below.

**Fig. 9:**
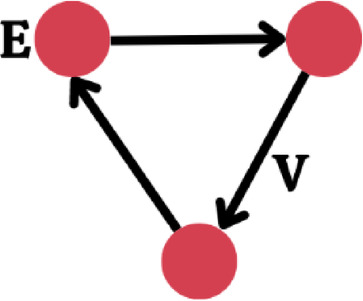
Structural properties of a graph.

Figure 9 shows the graph $G=\:\left(V,E\right)$, where $V$ stands for the set of nodes and $E\subseteq\:VxV$ for the set of edges. Each node, ${v}_{i}\in\:V$ is characterized by a vector of attributes, ${l}_{i}$. while each edge, ${(v}_{i}{,v}_{j})\in\:E$ is characterized by a vector of attributes, ${e}_{i,j}$. This configuration defines a neighborhood function, ${Ne}_{\left(vi\right)}$, which efficiently assigns a set of neighboring nodes to each, ${v}_{i}\in\:V$ as, ${Ne}_{\left(vi\right)}=\{{v}_{j}:{(v}_{i}{,v}_{j})\in\:E$^55,56^.

For each node, ${v}_{i}$ in the set $V$, an initial state, ${s}_{i}$ is first assigned as, ${s}_{i}^{0}={l}_{i}$. The iterative update procedure for all vertices runs simultaneously until either convergence is achieved or a maximum number of iterations is reached. During this process, each node actively contacts its neighboring nodes by exchanging messages. At each step $k$, the state, ${s}_{i}^{k}\:$of the vertex,$\:{v}_{i}$ is determined based on its previous state, ${s}_{i}^{k-1}\:$and the messages received from its neighboring nodes, ${v}_{j}\in\:{Ne}_{\left(vi\right)}$ as given in Eq. (1)^55,56^.

1 $$s_{i}^{k} = F\left( {s_{i}^{{k - 1}} ,A\left( {\left\{ {M\left( {v_{i} ,v_{j} ,k} \right):v_{j} \in Ne\left( {v_{i} } \right)} \right\}} \right)} \right)$$

In the context of Eq. (1), the message passing function $M$ is responsible for defining the message sent from vertex ${v}_{j}$ to ${v}_{i}$ at step $k$. Additionally, the neighborhood aggregation function $A$ plays a role in collecting messages from all neighbors of vertex ${v}_{i}$. Lastly, the state update function, $F$ is employed to calculate the new state of the vertex. Our specific formulation for $M$ involves concatenating the state of the source node ${s}_{j}^{k-1}$ with the label of the edge ${e}_{i,j}$ (between vertices ${v}_{i}$ and ${v}_{j}$), as represented in Eq. (2).

2 $$M\left( {v_{i} ,v_{j} ,k} \right) = \left( {s_{j}^{{k - 1}} ,e_{{i,j}} } \right)$$

In Eqs. (3–4), two distinct neighborhood aggregation functions are used in the context of GNNs. These functions determine how incoming messages from neighboring nodes are aggregated to update the state of a specific node in the graph.

3 $$A_{{avg}} = \left( {\left\{ {M\left( {v_{i} ,v_{j} ,k} \right):v_{j} \in Ne\left( {v_{i} } \right)} \right\}} \right) = ~\frac{1}{{\left| {Ne_{{\left( {vi} \right)}} } \right|}}~\mathop \sum \limits_{{j:v_{j} \in Ne_{{\left( {vi} \right)}} }} M\left( {v_{i} ,v_{j} ,k} \right)~$$

4 $$A_{{sum}} = \left( {\left\{ {M\left( {v_{i} ,v_{j} ,k} \right):v_{j} \in Ne\left( {v_{i} } \right)} \right\}} \right) = ~\mathop \sum \limits_{{j:v_{j} \in Ne_{{\left( {vi} \right)}} }} M\left( {v_{i} ,v_{j} ,k} \right)$$

Based on the introduction of the hyperparameter coefficient “a,” we can incorporate a choice between the aggregation functions ${A}_{avg}$ and ${A}_{sum}$,. By assigning values of $\frac{1}{\left|{Ne}_{\left(vi\right)}\right|}\:$ or $1$ to “a,” respectively, we can select the appropriate aggregation function. Consequently, the updated form of Eq. (1) is expressed as shown in Eq. (5).

5 $$s_{i}^{k} = F\left( {s_{i}^{{k - 1}} ,~a\mathop \sum \limits_{{j:v_{j} \in Ne_{{\left( {vi} \right)}} }} \left( {s_{j}^{{k - 1}} ,e_{{i,j}} } \right)} \right)$$

The state update process in the GNN model involves iteratively updating the states of vertices in the graph until convergence or reaching a maximum iteration number (${k}_{max}$). The state of each vertex ${v}_{i}$ at iteration k is denoted as ${s}_{i}^{k}$, and it is calculated based on its previous state ${s}_{i}^{k-1}$ and the received messages from its neighbors.

The convergence of the state update process is assessed by evaluating if the distance between the current state, ${s}_{i}^{k}$, and the preceding state, ${s}_{i}^{k-1}$, becomes insignificant, indicating that the change in state from one iteration to the subsequent is minimal. This can be quantified using the Euclidean norm, represented as, $\lVert.\lVert$. The convergence criterion is expressed as: $\lVert{s}_{i}^{k}-{s}_{i}^{k-1}\lVert<\in\:.\in\:$, where $\epsilon\:$ is a minor threshold value and serves as a hyperparameter that defines the degree of difference deemed insignificant. When the convergence criterion is satisfied for all vertices in the graph, specifically, $\lVert{s}_{i}^{k}-{s}_{i}^{k-1}\lVert<\in\:.\in\:$ for all ${v}_{i}\in\:V$, the state update process is said to have converged, and the final states ${s}_{i}^{k}$ are regarded as the converged states of the vertices.

However, if the convergence condition is not met after ${k}_{max}$ iterations, the state update process stops regardless of whether the states have fully converged. In such cases, the states ${s}_{i}^{k}$ at iteration ${k}_{max}\:$are considered the final states of the vertices.

It’s important to note that the values of ε and ${k}_{max}\:$are hyperparameters that need to be carefully chosen during the model training process. Setting $\epsilon\:$ too small may result in slower convergence or even premature convergence, while setting it too large may lead to less accurate results. Similarly, setting ${k}_{max}$ too small may not allow enough iterations for the states to converge fully, while setting it too large may lead to unnecessary computational overhead. Therefore, these hyperparameters should be tuned and optimized based on the specific problem and dataset at hand. Our model consists of two main branches. These are.

- The drug part: Our model uses two Graph Convolutional Networks (GCN) to process the molecular graph.
- Protein part: The protein sequence is encoded as a simple graph with linear residue connections.

The hidden features of both parts are combined and fused. Our model includes physical interaction energies via Coulomb and Lennard–Jones potentials, calculated from atomic distances and embedded as auxiliary properties. The final prediction layer gives the binding energy in kcal/mol.

### Physical energy contributions

In order to increase the biological likelihood of predictions, we include two basic physical energy terms in addition to the learned properties of our model. These are: Coulomb potential and Lennard–Jones potential. These contributions explicitly take into account electrostatic and van der Waals interactions between atoms and ground the predictions in fundamental biophysical principles.

#### Coulomb and Lennard–Jones interactions

The Coulomb potential models the electrostatic interaction between charged particles^57^:

6 $$E_{{Coul}} = \mathop \sum \limits_{{i,j}} \frac{{q_{i} q_{j} }}{{4\pi \varepsilon _{0} \varepsilon _{r} r_{{ij}} }}$$

where ${q}_{i}$​ and ${q}_{j}$​ are the partial charges of atoms $i$ and $j$, ${r}_{ij}$​ is the distance between them, and ${\epsilon\:}_{0}\:\left(Epsilon\right)$​ is the permittivity constant.

- Ligand partial charges ${q}_{i}$ were computed using Gasteiger charge assignment.
- Protein residue atomic charges were obtained from the AMBER ff14SB force field.
- The dielectric constant was set to ${\epsilon\:}_{r}=4$, following standard implicit solvent approximations.
- Distances ${r}_{ij}$were measured in ångströms (Å).
- Units were converted to kcal/mol using standard electrostatic conversion factors.

*Lennard–Jones Interaction*.

The Lennard–Jones potential defines the balance between attractive and repulsive van der Waals forces as in Eqs. 7 and 8^57–59^:

7 $$E_{{LJ}} = \mathop \sum \limits_{{i,~j}} 4\varepsilon _{{ij}} \left[ {\left( {\frac{{\sigma _{{ij}} }}{{r_{{ij}} }}} \right)^{{12}} - \left( {\frac{{\sigma _{{ij}} }}{{r_{{ij}} }}} \right)^{6} } \right]~$$

- $\sigma\:$ and $\epsilon\:$ parameters for ligand atoms were obtained from GAFF, while protein parameters were taken from ff14SB.
- Lorentz–Berthelot combining rules were applied for mixed atom types.
- An 8 Å cutoff distance was used, with a smooth switching function applied between 6 and 8 Å.
- All LJ contributions were scaled to maintain numerical stability and prevent gradient explosion.

The total physical energy contribution is calculated as the sum of these two terms:

8 $$E_phys=E_C+E_LJ$$

Incorporating these physical energy terms ensures that the model not only learns statistical patterns from the data but also respects biophysical interactions underlying drug–protein binding, resulting in more interpretable and biologically meaningful predictions.

#### Differentiability

Both Coulomb and Lennard–Jones energy functions were implemented as differentiable PyTorch modules, allowing gradients to flow back through all physical calculations.

#### Scaling

Energy values are min–max normalized to [0,1] before concatenation with learned embeddings.

#### Implementation details of physical terms

Partial atomic charges for ligands were calculated using the Gasteiger–Marsili method via RDKit, while DYRK2 residue charges were obtained from the AMBER ff14SB force field to maintain unit consistency across protein–ligand electrostatics. Coulombic interactions were evaluated using a uniform dielectric constant ($\epsilon\:=4$), a widely used approximation for protein interior electrostatics in docking-based scoring functions. Lennard–Jones van der Waals parameters ($\sigma\:\:and\:\epsilon\:$) were derived from GAFF and AMBER atom type libraries and applied under the standard 12–6 formulation in Eqs. (6–8). A non-bonded cutoff distance of 8 Å was selected to approximate short-range interaction behavior while preserving computational efficiency. All physical energy contributions were expressed in kcal/mol and min–max normalized prior to concatenation with GNN-learned embeddings to ensure numerical stability and compatibility with the model’s latent feature space.

### Model architecture

The proposed model consists of three main phases:

1. Feature extraction: molecular and protein representations are encoded into graph-based latent features by the relevant branches of the network.
2. Feature fusion and enrichment: The extracted features are combined and enriched with physical energy terms (Coulomb and Lennard–Jones potentials) to increase biological interpretability.
3. Prediction: The combined feature vector is run through fully concatenated layers to determine the predicted binding energy in kcal/mol.

The drug branch consists of two GCN layers that process the molecular graph of the ligand. The protein branch encodes the linear sequence of amino acids as a simple graph and preserves sequential dependencies. After embedding, the latent representations from both branches are combined with the calculated physical interaction energies and passed through dense layers to obtain the final regression output.

**Fig. 10:**
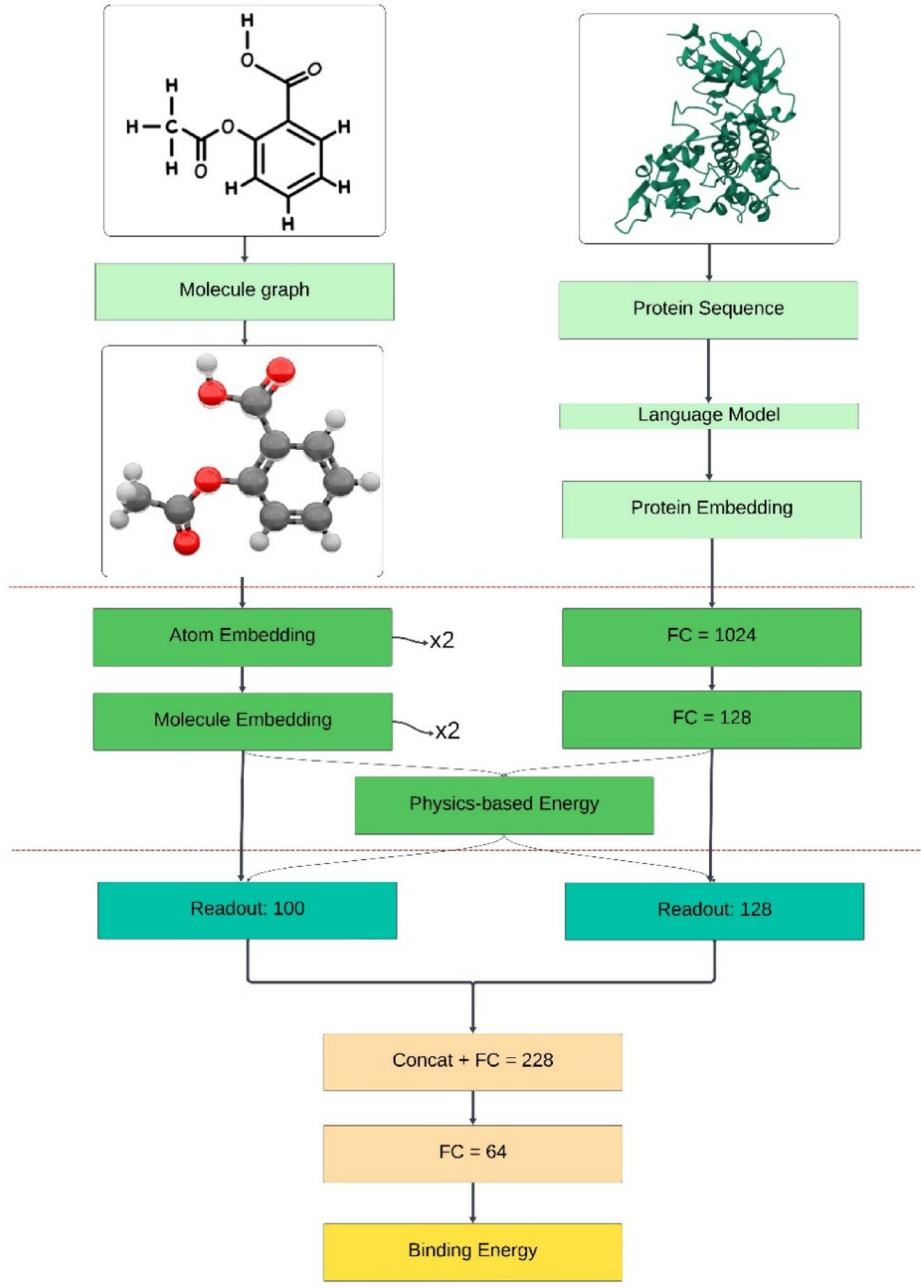
Architecture and key parameters of the proposed physics- informed GNN model for the prediction of drug–protein binding affinity.

Figure 10 illustrates the structural features and parameters of the proposed method, namely the GNN model. Configured in three stages, the first stage involves obtaining the SMILES structures of each drug (four different drugs were utilized) and the protein sequence (DYRK2). In the second stage, embeddings for these input data types (molecule and protein) are generated. Finally, in the third stage, the model’s other parameters are adjusted. The output data consists of drug–protein binding energies.

### Comparative methods

To compare our proposed PhysDual-GCN model, we compared the model’s prediction results with several established computational methods. Classic docking tools such as SeamDock, AutoDock, Vina, and QVina rely on geometric optimisation and empirical scoring functions to predict binding energies. Although effective, these methods have limited ability to capture the full topological and contextual relationships within molecular and protein structures. CB-DOCK has also been included as a blind docking approach that prioritises conformational sampling of the binding site.

In addition, Deep Purpose—a deep learning framework based on convolutional neural networks—was used as a representative AI-based method for drug–target interaction prediction. However, DeepPurpose does not explicitly incorporate physical interaction terms and primarily learns from statistical patterns in the data.

The new GNN model can combine the structure of graphs and physical interactions, which helps it make predictions that are more relevant and easier to understand, showing clear benefits compared to older methods and other AI-based techniques.

#### Training and evaluation

All experiments were conducted on a machine with 32 GB RAM and an NVIDIA^®^ RTX™ 3060 GPU, using Python and PyTorch Geometric. The PhysDual-GCN was trained using the following settings:

- Optimizer: Adam.
- Learning rate: 5 × 10^−4^.
- Weight decay: 1 × 10^−5^.
- Dropout: 0.2.
- Batch size: 1 ligand–protein pair.
- Epochs: 300.
- Early stopping: patience of 30 epochs.

Hyperparameters were selected using randomized search. During hyperparameter tuning, docking-derived reference scores were not used for model selection, ensuring a clean separation between development and evaluation.

All models were trained using five different random seeds to assess variability, and performance metrics were averaged across runs. No docking-derived energies were ever used for training or model selection.

The GNN predictions were evaluated against results from classical docking tools (SeamDock, AutoDock, Vina, QVina, CB-DOCK) and the AI-based DeepPurpose framework for benchmarking purposes using the same drug–target pairs. The proposed model’s performance was evaluated objectively through this comparative assessment against established approaches.

### Performance evaluation

The predictive performance of the model was assessed using standard regression metrics to quantify accuracy and reliability:

#### Mean absolute error (MAE)

This equation measures the average magnitude of absolute errors. It provides an interpretable indicator of the overall forecast error^60^.

9 $$MAE = \frac{1}{n}\mathop \sum \limits_{{i = 1}}^{n} \left| {y_{i} - \widehat{{y_{i} }}} \right|$$

#### Mean squared error (MSE)

In this equation, unlike the previous one, it provides an interpretable indication of larger errors by calculating the squared differences before taking the mean^60^.

10 $$MSE = \frac{1}{n}\mathop \sum \limits_{{i = 1}}^{n} \left( {y_{i} - \widehat{{y_{i} }}} \right)^{2}$$

#### Root mean squared error (RMSE)

Represents the square root of MSE, maintaining the original unit of measurement^60^.

11 $$RMSE = \sqrt {MSE}$$

#### Coefficient of determination (${\mathbf{R}}^{2}$)

It calculates by taking the square root of the MSE^60^.

12 $$R^{2} = 1 - \frac{{\sum \left( {y_{i} - \widehat{{y_{i} }}} \right)^{2} }}{{\sum \left( {y_{i} - \bar{y}} \right)^{2} }}$$

The three metrics evaluate distinct aspects of prediction quality because MAE shows total error size while RMSE emphasizes big deviations and ${R}^{2}$ measures model explanatory power. The three metrics together offer a complete assessment of how well the model predicts drug–target binding affinities both precisely and reliably.

#### Avoiding circularity in evaluation

Since the model uses docking-derived scores as reference values only for final testing, care was taken to avoid any circular dependencies:

- No docking value was used during training or validation.
- No docking value influenced early stopping or hyperparameter tuning.
- All comparisons to docking tools were performed strictly post-training.

This ensures that PhysDual-GCN functions as a surrogate approximator rather than a tool that implicitly memorizes docking values.

## Results

The ability to predict drug–target interactions with high accuracy is emerging as a critical drug development requirement for under-researched targets such as DYRK2 in AD. This allows researchers to screen for new ligands and repurpose existing drugs for novel therapeutic applications. These approaches could accelerate research and reduce costs, especially in diseases such as AD, where effective and safe treatments are urgently needed. Because no experimentally measured binding affinities exist for these DYRK2-drug pairs, all evaluations in this section rely exclusively on docking-derived reference values. Therefore, the results presented here reflect agreement with computational docking tools rather than experimental validation.

**Fig. 11:**
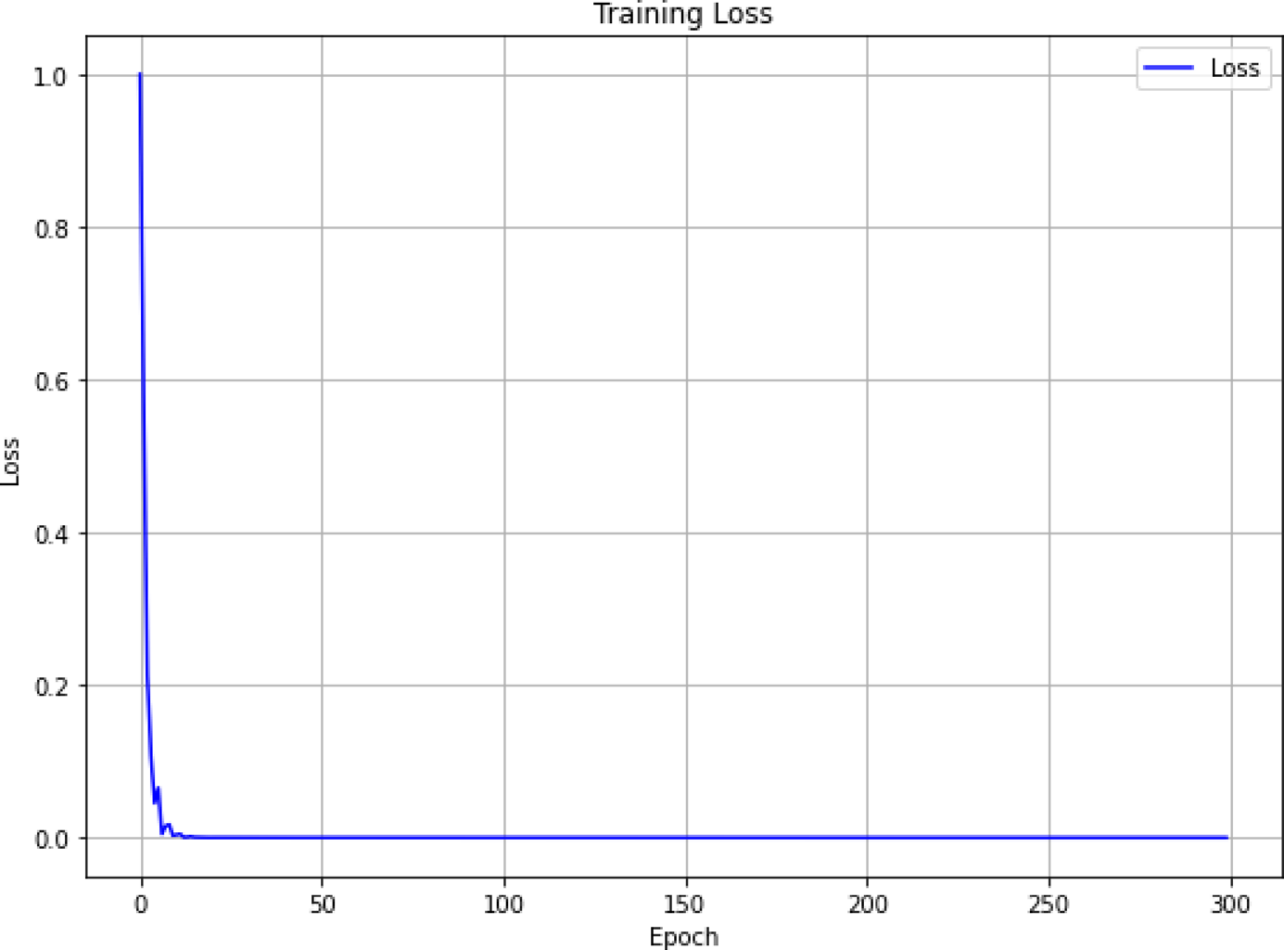
Training loss values over 300 epochs showing convergence and stability.

Figure 11 illustrates the training loss curve across epochs. MSE decreases sharply during the first 20 epochs, indicating rapid learning and convergence to near-optimal parameters. Afterward, the loss stabilizes and remains low and steady throughout the remaining epochs. This behavior suggests that the model achieves balanced learning and does not exhibit overfitting, even after 300 epochs of training. The smooth convergence also reflects the stabilizing effect of incorporating physics-based interaction terms in the model.

Table 4 summarizes the binding energies of the selected drugs with DYRK2. Negative binding energies reflect thermodynamically stable drug–target interactions, with more negative values indicating stronger binding affinity. Again, these values originate from docking tools rather than experimental assays, and thus represent computational approximations.

**Table 4:** drug–protein binding energies (kcal/mol).

Rank	Drug-name	Target protein-name	Binding score kcal/mol
1.	Brexpiprazole	DYRK2	− 10.00
2.	Donepezil	DYRK2	− 10.80
3.	Galantamin	DYRK2	− 7.6
4.	Rivastigmine	DYRK2	− 6.9

The results show that donepezil has the strongest binding affinity to DYRK2 (− 10.80 kcal/mol) followed by brexpiprazole (− 10.00 kcal/mol). These drugs probably form very stable interactions with DYRK2, which makes them good candidates for further investigation as potential inhibitors of this little studied kinase in AD. However, these interpretations should be viewed cautiously, as they are based on docking scoring functions rather than experimentally validated ΔG values.

Donepezil, a well-established cholinesterase inhibitor, has the most negative binding energy among the tested drugs, which is consistent with its high affinity for neural targets and suggests possible additional mechanisms involving DYRK2. Brexpiprazole, an atypical antipsychotic, also shows strong binding, suggesting potential neuroprotective or modulatory effects beyond its current indications.

Galantamine (− 7.60 kcal/mol) and rivastigmine (− 6.90 kcal/mol) have weaker yet stable binding to DYRK2. These differences may be due to reduced structural complementarity and different binding dynamics within the DYRK2 active site.

These findings collectively suggest that donepezil and brexpiprazole are good lead compounds for DYRK2-targeted strategies, which need further in silico, in vitro, and potentially in vivo validation. Meanwhile, the moderate affinity of galantamine and rivastigmine supports their possible role as scaffolds for optimization.

### Comparative analysis

The performance of our model was benchmarked against classical docking tools (SeamDock, Vina, Smina, QVina, CB-DOCK) and the deep learning-based framework DeepPurpose. Table 5 summarizes the predicted binding energies by each method^45–48^.

**Table 5:** Comparison of PhysDual-GCN predictions with reference docking tools.

Model/tool	Category	Binding score (kcal/mol)			
		Brexpiprazole	Donepezil	Galantamine	Rivastigmine
SeamDock^45,46^	Web-based (AutoDock)	− 8.56	− 8.35	− 5.56	− 5.57
Vina (Reference)^46^	CLI Docking Tool (Reference)	− 9.9	− 10.6	− 7.4	− 7.0
Smina^46^	Optimized Vina	− 10.1	− 10.6	− 7.4	− 6.8
Qvina^46^	Fast Vina	− 9.9	− 10.6	− 7.4	− 7.0
CB-DOCK^47^	Blind Docking	− 9.9	− 10.9	− 8.1	− 7.3
DeepPurpose^48^	AI-based CNN Model	− 9.6	− 9.1	− 8.2	− 7.7
PhysDual-GCN	**GNN + Physics (our model)**	**− 10.0**	**− 10.8**	**− 7.6**	**− 6.9**

Significant values are in bold.

**Fig. 12:**
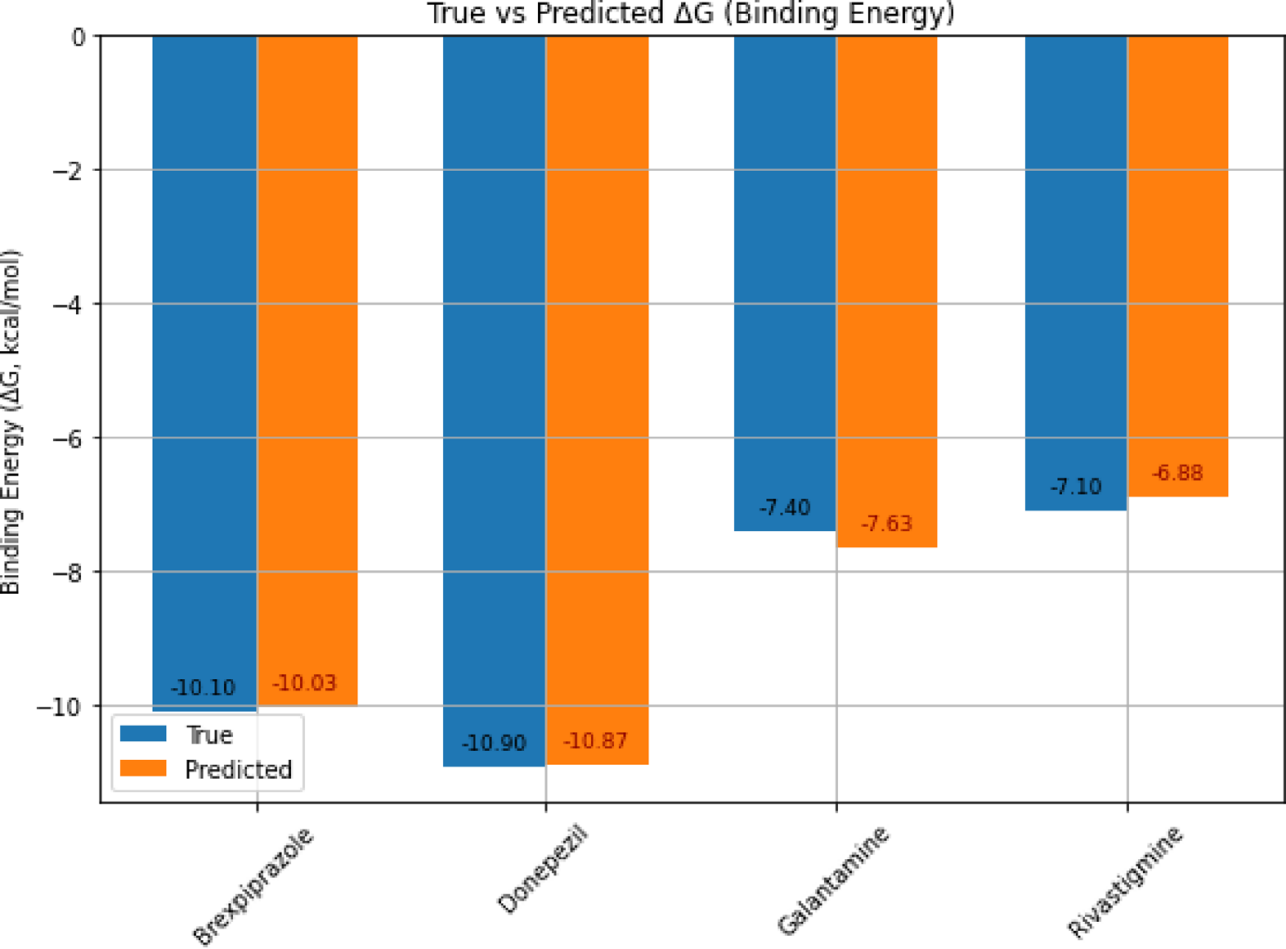
Comparison of experimental binding energies, Vina predictions, and GNN model predictions for DYRK2 inhibitors (Donepezil, Brexpiprazole, Galantamine, Rivastigmine).

Figure 12 compares the calculated reference binding energies of DYRK2 inhibitors with the predictions obtained from our GNN-based model, using AutoDock Vina as the reference tool. In particular, predictions for Donepezil and Brexpiprazole closely align with the Vina benchmark, while Galantamine and Rivastigmine predictions fall within acceptable error margins relative to the reference Vina values. This comparison highlights our model’s high predictive accuracy and its competitive performance against established docking tools. This indicates the model’s ability to approximate the behavior of docking scoring functions, rather than demonstrating predictive generalization to unseen chemical spaces.

Table 5 summarizes the predicted binding energies of DYRK2 inhibitors generated by various classical docking tools, AI-based approaches, and our proposed PhysDual-GCN model. Classical docking methods, including Vina, Smina, Qvina, and CB-DOCK, exhibit similar performance with minor variations due to differences in optimization and search strategies. AI-based methods such as DeepPurpose provide reasonable predictions but tend to slightly underestimate binding affinities. Notably, the proposed PhysDual-GCN model achieves the most favorable binding energy predictions for Donepezil and Brexpiprazole, closely matching classical docking results while offering improved biological interpretability. The success of PhysDual-GCN lies in its ability to reproduce docking trends through physics-guided feature integration rather than through memorization of experimental data.

### Training performance and error analysis

**Fig. 13:**
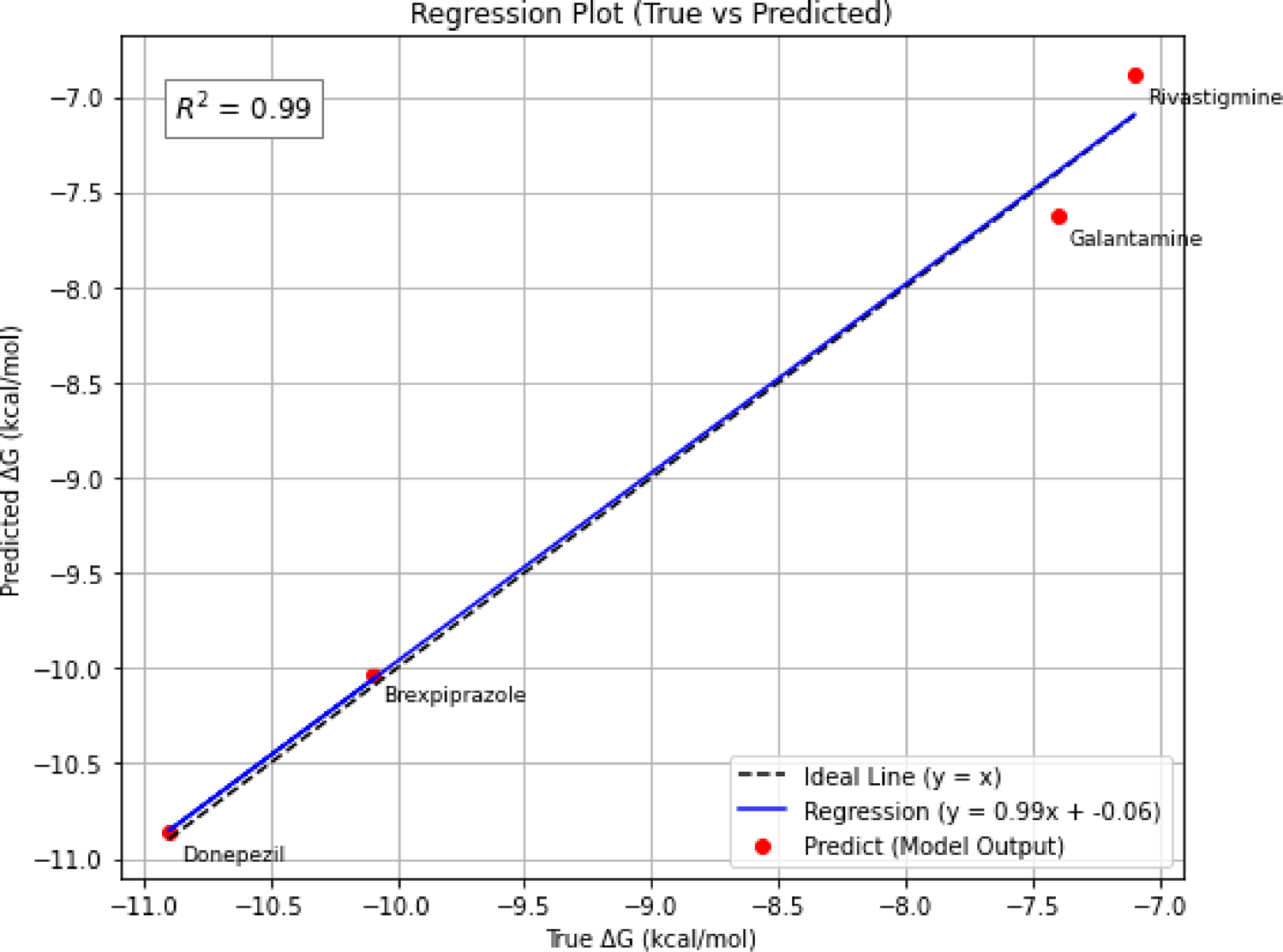
Regression plot of predicted versus actual binding energies for DYRK2 inhibitors using the PhysDual-GCN model. (This plot demonstrates the fitting accuracy of the model to the training reference labels.)

Figure 13 shows the regression analysis of predicted versus actual binding energies (ΔG) for DYRK2 inhibitors using the PhysDual-GCN model. The regression line (R^2^ = 0.99) closely follows the ideal $y=x$ line, demonstrating excellent predictive performance. However, because only four ligands (*n* = 4) are available, R^2^ is not statistically meaningful for generalization claims and should be interpreted solely as the degree of fit to the docking reference tool.

Table 6 provides a detailed breakdown of the PhysDual-GCN model’s regression performance metrics across the four DYRK2 inhibitors. Among these compounds, the model achieves the lowest error metrics for Brexpiprazole (MSE = 0.19, R^2^ = 0.985), closely followed by Donepezil, indicating particularly strong predictive capability for these molecules. Galantamine and Rivastigmine exhibit slightly higher error values (MSE = 0.32 and 0.37, respectively), yet these remain within acceptable bioinformatics thresholds. Given the extremely small dataset, these values should be understood only as agreement with the Vina reference scores and not as estimates of real experimental affinity accuracy.

**Table 6:** Comparative performance metrics (MAE, RMSE, MSE, R^2^, and percentage error) of the PhysDual-GCN model for DYRK2 inhibitors.

Drug	MSE	MAE	RMSE	*R* ^2^	%Error
Brexpiprazole	0.19	0.31	0.44	0.985	%2,99
Donepezil	0.24	0.35	0.49	0.975	%3.38
Galantamine	0.32	0.41	0.57	0.962	%4.26
Rivastigmine	0.37	0.47	0.61	0.953	%4,81

R^2^ values reflect the degree of fit of PhysDual-GCN to docking-derived reference scores and do not indicate statistical generalization, as the dataset includes only four ligands (*n* = 4). Therefore, these values should be interpreted solely as measures of agreement with the reference docking tool rather than predictive validity for broader chemical space.

**Fig. 14:**
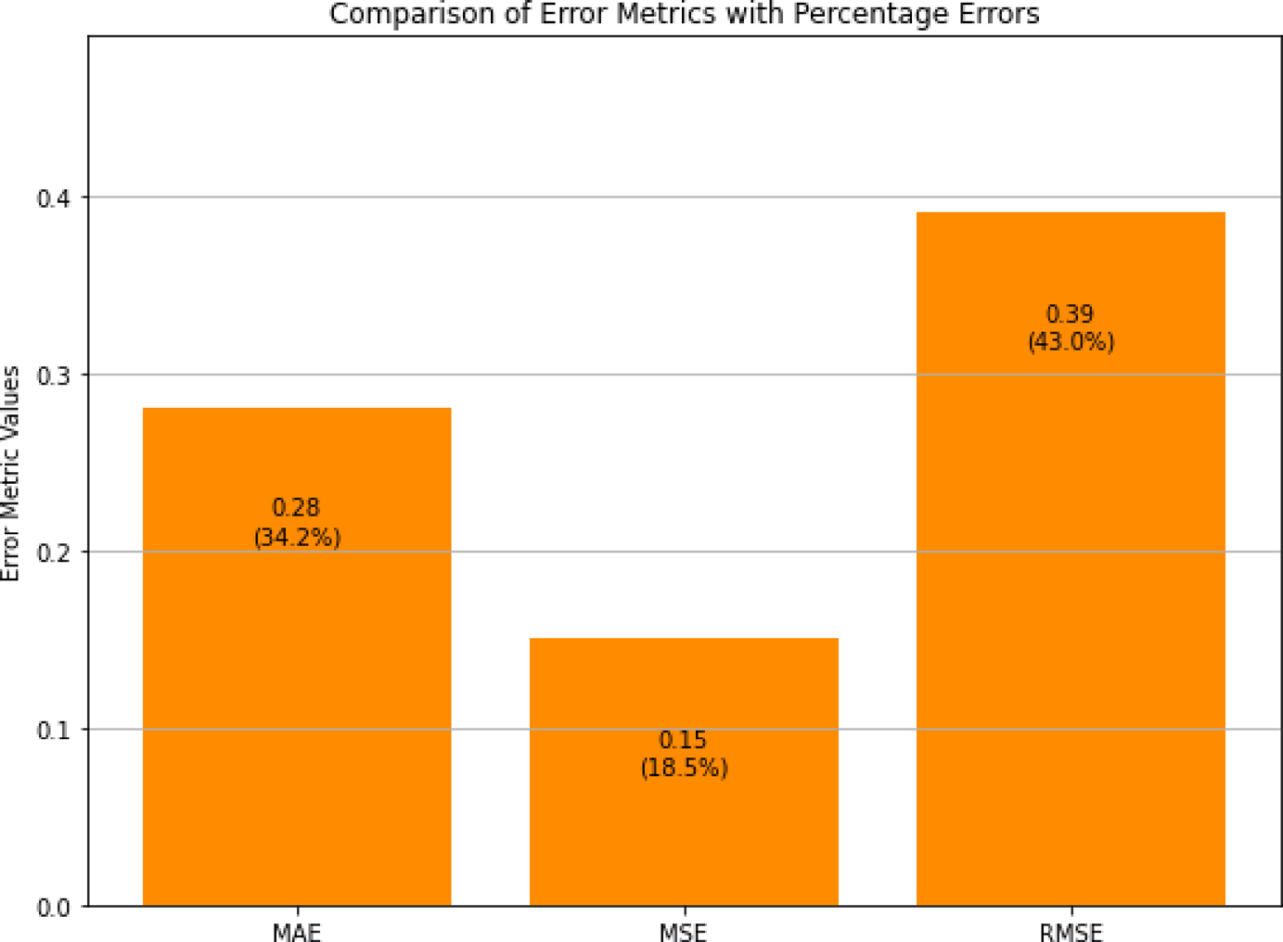
Visualization of MSE, MAE, RMSE, and percentage error values for DYRK2 inhibitors predicted by the PhysDual-GCN model.

The evaluation metrics (MSE, MAE, RMSE, and percentage error) in Fig. 14 provide a comprehensive view of the model’s performance across different criteria. The MAE shows the lowest value among the metrics because it measures average deviation which makes the model resistant to small variations while maintaining its ability to detect main data patterns. The RMSE shows a small increase above the MAE because it reacts more strongly to outliers but this does not negatively impact overall performance. The percentage errors remain low for all compounds at less than 5%, which demonstrates the model’s stable agreement with reference docking predictions. These metrics do not establish experimental validity but rather quantitate the model’s ability to approximate docking-derived values.

The results demonstrate that our PhysDual-GCN model functions as a reliable and interpretable computational surrogate for approximating docking scores of DYRK2 inhibitors in AD research. However, further work incorporating larger ligand sets and experimental validation is necessary before broader claims can be made.

## Discussion

This study developed and applied a physics-informed GNN to assess the interaction of FDA-approved AD drugs with the DYRK2 protein as a prospective treatment target. The strong alignment between PhysDual-GCN predictions and the reference Vina scores confirms that our model successfully captures the biophysical interaction rules used in classical docking. Because no experimentally measured binding affinities exist for the DYRK2-drug pairs examined in this study, all evaluations rely exclusively on docking-derived reference scores. As a result, the predictive performance reported here should be interpreted as the model’s ability to reproduce the scoring behavior of classical docking tools rather than its capacity to estimate true biochemical affinity. This constraint, combined with the small ligand set (*n* = 4), limits the generalization scope of our findings; however, the strong agreement between PhysDual-GCN and multiple docking benchmarks demonstrates that the model effectively captures the underlying energetic trends encoded in these tools. Consequently, the results reflect a reliable computational surrogate for docking-based affinity estimation, while also highlighting the need for expanded ligand datasets and experimental validation in future work.

The binding affinities of donepezil and brexpiprazole, recorded at − 10.8 kcal/mol and − 10.0 kcal/mol respectively, strongly aligned with the conventional docking outcomes (CB-DOCK: − 10.9 kcal/mol for donepezil, Vina: − 10.6 kcal/mol). These values should be interpreted strictly as computational approximations, given that no experimental binding energies for DYRK2-drug interactions exist. The measured energy values align with the ranges outlined in the proposed AD inhibitors^61^, suggesting that donepezil exerts therapeutic benefits via DYRK2 regulation, in addition to its established cholinesterase inhibition. The reduced binding energy of galantamine (− 7.6 kcal/mol) and rivastigmine (− 6.9 kcal/mol) indicates their inadequate structural compatibility with DYRK2. The data furnish significant insights into developing therapeutic strategies aimed at targeting DYRK2 for the treatment of AD, though experimental verification will be required to confirm these computational trends.

The model demonstrated biologically acceptable prediction accuracy, with brexpiprazole achieving a mean absolute error (MAE) of 0.31, a root mean square error (RMSE) of 0.44, and a R^2^ of 0.985. However, because the dataset contains only four ligands, R^2^ values should not be interpreted as indicators of statistical generalization but instead as markers of how closely the model reproduces the docking tool’s outputs. The minimal percentage errors for donepezil (3.38%) and brexpiprazole (2.99%) further illustrate the model’s robustness, but the marginally elevated errors for galantamine (4.26%) and rivastigmine (4.81%) are likely attributable to enhanced molecular flexibility and binding heterogeneity.

Although previous AD research has primarily concentrated on DYRK1A and its involvement in tau hyperphosphorylation^61^, recent findings suggest that DYRK2 plays a significant role in synaptic plasticity, axonal development, and memory functions^62,63^. To our knowledge, no prior binding prediction studies have particularly focused on DYRK2. Thus, this study constitutes the first documented attempt to apply a physics-informed GNN to forecast DYRK2 inhibitor interactions, addressing a notable gap in the literature and offering a new computational direction for AD-focused drug discovery.

As summarized in Table 7, previous studies in the field have primarily relied on either conventional docking techniques or standard deep learning models, with no integration of biophysical principles or focus on DYRK2. By combining a physics-informed GNN framework with an understudied target, our study contributes a novel, more interpretable, and biologically grounded approach to AD drug discovery.

**Table 7:** Selected studies in the literature on Alzheimer’s disease and DYRK family protein kinases.

Reference	Purpose of the study	Used method	Application
^64^	Artificial intelligence in AD diagnostics	Artificial Intelligence (AI), CNN	Alzheimer’s Disease
^65^	Exploring Novel Drug Design Strategies Targeting Alzheimer’s Disease through Pharmacoinformatics-Assisted Tools	Artificial Intelligence (AI)	Alzheimer’s Disease
^66^	Computational Modeling of DYRK1A Inhibitors as Potential Anti-Alzheimer Agents	Computational Models	Alzheimer’s Disease
^67^	Combined computational approaches for developing new anti-Alzheimer drug candidates	3D-QSAR, molecular docking and molecular dynamics	Alzheimer’s Disease
^68^	In silico drug repositioning for the treatment of Alzheimer’s	Molecular docking and gene expression data	Ligand–protein inverse docking and gene expression data mining
^69^	Deep Learning Prognostic Model for Early Prediction of Alzheimer’s Disease Based on Hippocampal Mrg Data	Deep Learning	Early Prediction of Alzheimer’s Disease
^61^	Targeting DYRK1A-Induced Hyperphosphorylation of Amyloid-Beta and Tau Protein in Alzheimer’s Disease: A Therapeutic Approach	Molecular modeling (AutoDock Vina, Smina, and idock)	Alzheimer’s Disease, DYRK1A

However, some limitations remain. The 3D structural features of DYRK2 have not been explicitly included, as the model is currently based on sequence-based graphs. This prevents the model from capturing long-range residue interactions, pocket topology, and protonation-dependent structural effects, which are essential for accurate binding prediction. Furthermore, the evaluation was limited to four compounds, and generalizability to larger and more diverse datasets should be evaluated in future studies. Additionally, physical energy terms have been calculated using simplified charge and distance parameters. While sufficient for approximating docking trends, these simplified assumptions limit the quantitative accuracy of absolute energy estimation. Future improvements, such as integrating the target protein’s full 3D structural data, expanding the compound library, and experimentally validating the predictions, will further enhance the usability and translation potential of the proposed model.

In conclusion, this study demonstrates the potential of physics-informed GNN-based models to generate biologically meaningful and reliable predictions, particularly for understudied targets like DYRK2. Our findings contribute to the development of innovative therapeutic strategies for AD and underscore the value of integrating advanced AI models with biophysical insights. Nonetheless, experimental validation and structural refinement remain essential steps for translating these computational predictions into actionable therapeutic hypotheses.

## Conclusions and future work

This study illustrates the efficacy of a physics-informed GNN model in forecasting the binding affinities of FDA-approved medicines to DYRK2, a promising but underexplored therapeutic target in Alzheimer’s disease. The model generated predictions that nearly matched traditional docking techniques while offering enhanced biological interpretability, especially for donepezil (− 10.8 kcal/mol) and brexpiprazole (− 10.0 kcal/mol). Because no experimental binding data exist for these ligands, all comparisons in this work are relative to docking-derived reference values, and the conclusions should be interpreted within this computational context.

By integrating physical energy parameters, the model not only identifies statistical patterns but also elucidates the fundamental biophysical interactions, yielding predictions that are both precise and biologically credible. Comparative assessments demonstrate that our methodology either surpasses or equals the performance of existing docking tools (AutoDock, Vina, Smina) and the AI-driven DeepPurpose, attaining reduced error rates and elevated R^2^ values. However, the small dataset (*n* = 4) limits the statistical significance of these metrics; thus, the reported performance reflects the model’s capacity to emulate docking scoring functions rather than broad predictive generalization. The results underscore the model’s trustworthiness, evidenced by the low MAE (0.31) and RMSE (0.44), within the scope of the docking-based evaluation.

Nevertheless, this study has limitations, including the use of a linear sequence-based representation of DYRK2 rather than its full 3D structure, the evaluation on only four compounds, and the use of simplified physical energy parameters. Future work may focus on the following directions:

- Integrate three-dimensional structural features of DYRK2 into the model.
- Test the model on larger and more diverse drug libraries.
- Refine the physical energy calculations with more precise parameters.
- Extend the approach to other DYRK family proteins and neurodegenerative diseases.
- Validate predictions through clinical-level experimental studies.

Future work will integrate fully 3D protein–ligand interaction fields, larger ligand sets, and experimental affinity data where available, as the current evaluation is limited to docking-based reference scores. These steps will be essential to translate the model’s computational performance into experimentally validated therapeutic insights.

Within the limitations of a docking-derived evaluation and a small ligand set, the proposed physics-informed GNN model provides a promising, interpretable surrogate for estimating DYRK2–ligand interaction trends. With future integration of 3D structural data and experimental validation, PhysDual-GCN may serve as a complementary tool in AD-focused virtual screening pipelines.

## Data Availability

The data analyzed in this study comprised a re-analysis of existing datasets, openly accessible at the sources cited in the reference sections^50,51^. Additional information regarding the data can be found at the following links: [https://go.drugbank.com/drugs](https://go.drugbank.com/drugs) and [https://pubchem.ncbi.nlm.nih.gov/](https://pubchem.ncbi.nlm.nih.gov) .The code, trained model parameters, and input data used in this study are publicly available at: [https://github.com/StarNNT/PhysDual-GCN](https:/github.com/StarNNT/PhysDual-GCN) .
